# Supplementary material for: Neurovascular coupling preserved in a chronic mouse model of Alzheimer’s disease: Methodology is critical
Source: J Cereb Blood Flow Metab. 2019 Nov 23;40(11):2289–303. doi: 10.1177/0271678X19890830 (PMC7585931; doi:10.1177/0271678X19890830)
Supplement: JCB890830 Supplemetal Material1 - Supplemental material for Neurovascular coupling preserved in a chronic mouse model of Alzheimer’s disease: Methodology is critical [file JCB890830_Supplemetal_Material1.pdf]

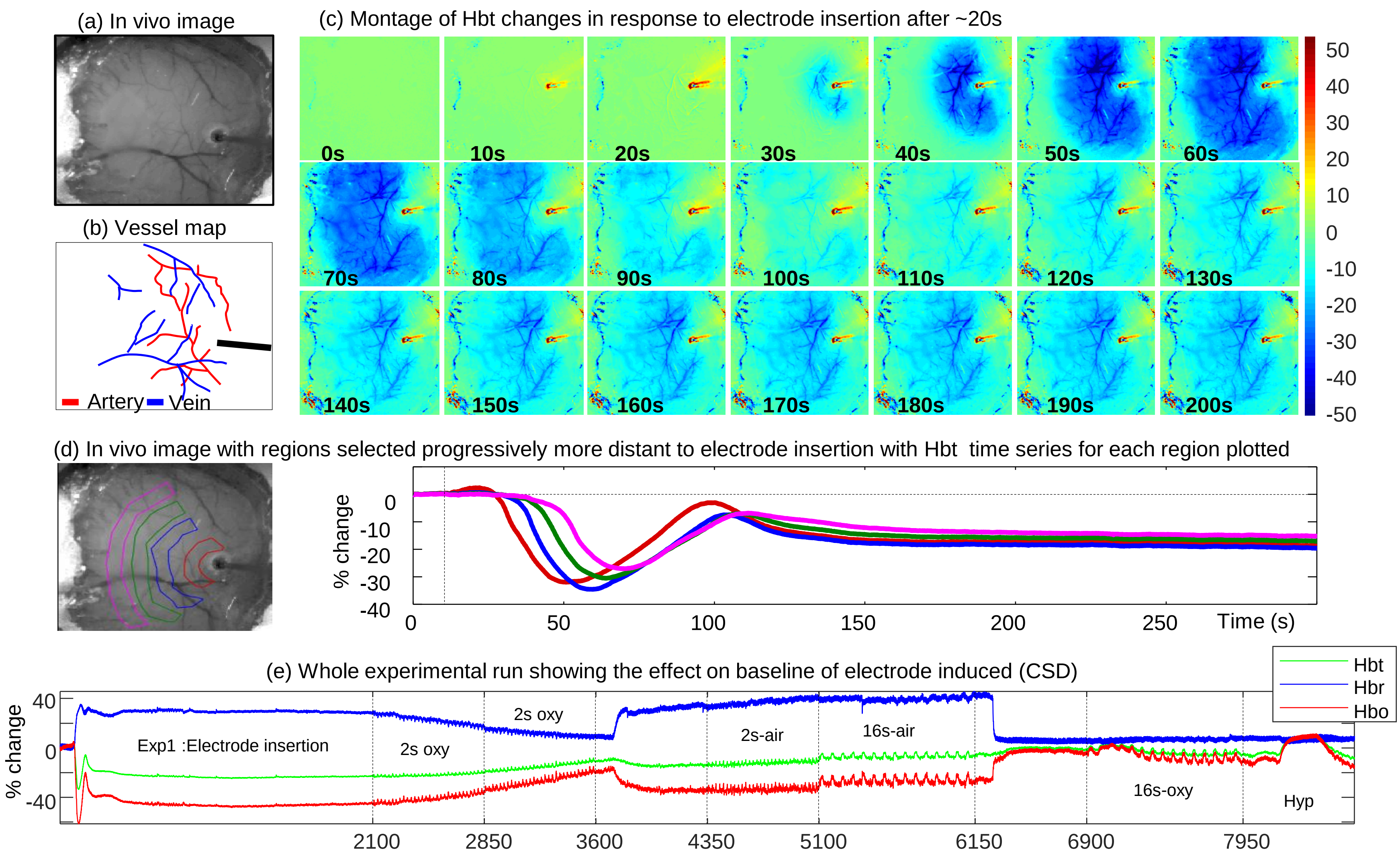

Supplementary Figure 1 : Cortical spreading depression (CSD) caused by electrode insertion in a 12 month J20-AD mouse. (a) Reference image showing electrode inserted into the cortex. (b) Vessel map showing location of surface arteries and veins. (c) Montage showing dynamic effect of electrode insertion after 20s, There is a wave of vasoconstriction that travels across the cortex. Each image is an average of 10s of data. (d) Concentric regions selected around electrode tip with corresponding Hbt time series from each region. (e) Whole experimental day showing the effect electrode insertion has on subsequent data collection.
