# Supplementary material for: Neurovascular coupling preserved in a chronic mouse model of Alzheimer’s disease: Methodology is critical
Source: J Cereb Blood Flow Metab. 2019 Nov 23;40(11):2289–303. doi: 10.1177/0271678X19890830 (PMC7585931; doi:10.1177/0271678X19890830)
Supplement: JCB890830 Supplemetal Material2 - Supplemental material for Neurovascular coupling preserved in a chronic mouse model of Alzheimer’s disease: Methodology is critical [file JCB890830_Supplemetal_Material2.pdf]

Supplementary Table 1: Two way ANOVA to assess changes in Hemodynamic responses between WT and J20-AD across chronic sessions.

| | Hbt Exp1 | Hbt Exp2 | Hbt Exp4 | Hbt Exp5 | Hbt Exp7 |
|---|---|---|---|---|---|
| Animal Type | F=0.001<br>p= 0.98 | F=0.001<br>p= 0.972 | F=0.094<br>p= 0.76 | F=0.341<br>p= 0.565 | F=0.037<br>p= 0.855 |
| Session(1:3) | F=4.748<br>p= 0.018 | F= 2.944<br>p= 0.072 | F= 3.117<br>p= 0.062 | F=1.636<br>p= 0.216 | F=5.885<br>p= 0.008 |
| Interaction | F=0.169<br>p=0.846 | F=0.123<br>p= 0.88 | F= 0.180<br>p= 0.836 | F=1.061<br>p= 0.361 | F=0.057<br>p= 0.944 |
| | Hbo Exp1 | Hbo Exp2 | Hbo Exp4 | Hbo Exp5 | Hbo Exp7 |
| Animal Type | F=0.018<br>p=0.89 | F=0.19<br>p= 0.662 | F=0.295<br>P=0.592 | F=2.198<br>p= 0.151 | F=0.308<br>p= 0.587 |
| Session(1:3) | F=5.478<br>p= 0.011 | F=4.434<br>p= 0.023 | F= 3.133<br>p= 0.0618 | F= 1.655<br>p= 0.212 | F=7.403<br>p= 0.003 |
| Interaction | F=0.341<br>p= 0.715 | F=0.084<br>p= 0.919 | F=0.0614<br>p= 0.941 | F=0.351<br>p= 0.707 | F=0.008<br>p= 0.992 |
| | Hbr Exp1 | Hbr Exp2 | Hbr Exp4 | Hbr Exp5 | Hbr Exp7 |
| Animal Type | F=0.042<br>p= 0.84 | F=1.211<br>p= 0.282 | F=1.691<br>p= 0.206 | F= 5.178<br>p= 0.032 | F=0.548<br>p= 0.466 |
| Session (1-3) | F=6.195<br>p= 0.007 | F=6.091<br>p= 0.007 | F=4.284<br>p= 0.026 | F=2.441<br>p=0.108 | F=2.891<br>p= 0.075 |
| Interaction | F=0.607<br>p=0.553 | F=0.512<br>p= 0.606 | F=0.463<br>p= 0.206 | F=0.871<br>p= 0.43 | F=0.121<br>p= 0.886 |

Supplementary Table 2: Average hemodynamic responses (with SD) for chronic and acute session experiments

| | Average Chronic Session | | | | | Acute Session (with Electrode) | | | | |
|---|---|---|---|---|---|---|---|---|---|---|
| | Exp1 | Exp2 | Exp4 | Exp5 | Exp7 | Exp1 | Exp2 | Exp4 | Exp5 | Exp7 |
| WT Hbt | 2.48 ± 0.23 | 1.99 ± 0.19 | 2.23 ± 0.32 | 2.81 ± 0.27 | 2.80 ± 0.17 | 0.76 ± 0.39 | 0.92 ± 0.42 | 1.41 ± 0.51 | 1.97 ± 0.52 | 1.99 ± 0.44 |
| AD Hbt | 2.49 ± 0.42 | 1.98 ± 0.31 | 2.19 ± 0.13 | 2.73 ± 0.11 | 2.77 ± 0.13 | 0.37 ± 0.08 | 0.58 ± 0.14 | 1.04 ± 0.23 | 1.55 ± 0.24 | 1.41 ± 0.40 |
| WT Hbo | 4.68 ± 0.20 | 3.83 ± 0.17 | 4.64 ± 0.29 | 5.81 ± 0.24 | 5.12 ± 0.20 | 1.38 ± 0.36 | 1.79 ± 0.39 | 2.82 ± 0.47 | 3.84 ± 0.42 | 3.61 ± 0.35 |
| AD Hbo | 4.62 ± 0.39 | 3.68 ± 0.28 | 4.48 ± 0.19 | 5.39 ± 0.16 | 4.99 ± 0.10 | 0.48 ± 0.06 | 0.99 ± 0.11 | 1.91 ± 0.20 | 2.78 ± 0.20 | 2.39 ± 0.34 |
| WT Hbr | -3.13 ± 0.39 | -2.71 ± 0.29 | -2.18 ± 0.54 | -2.36 ± 0.53 | -2.78 ± 0.42 | -0.86 ± 0.62 | -1.30 ± 0.69 | -1.33 ± 0.53 | -1.48 ± 0.42 | -1.90 ± 0.66 |
| AD Hbr | -3.04 ± 0.39 | -2.38 ± 0.28 | -1.86 ± 0.19 | -1.72 ± 0.16 | -2.55 ± 0.10 | -0.06 ± 0.10 | -0.59 ± 0.18 | -0.87 ± 0.42 | -0.89 ± 0.45 | -0.98 ± 0.44 |
