# Supplementary material for: Neurovascular coupling preserved in a chronic mouse model of Alzheimer’s disease: Methodology is critical
Source: J Cereb Blood Flow Metab. 2019 Nov 23;40(11):2289–303. doi: 10.1177/0271678X19890830 (PMC7585931; doi:10.1177/0271678X19890830)
Supplement: JCB890830 Supplemetal Material3 - Supplemental material for Neurovascular coupling preserved in a chronic mouse model of Alzheimer’s disease: Methodology is critical [file JCB890830_Supplemetal_Material3.pdf]

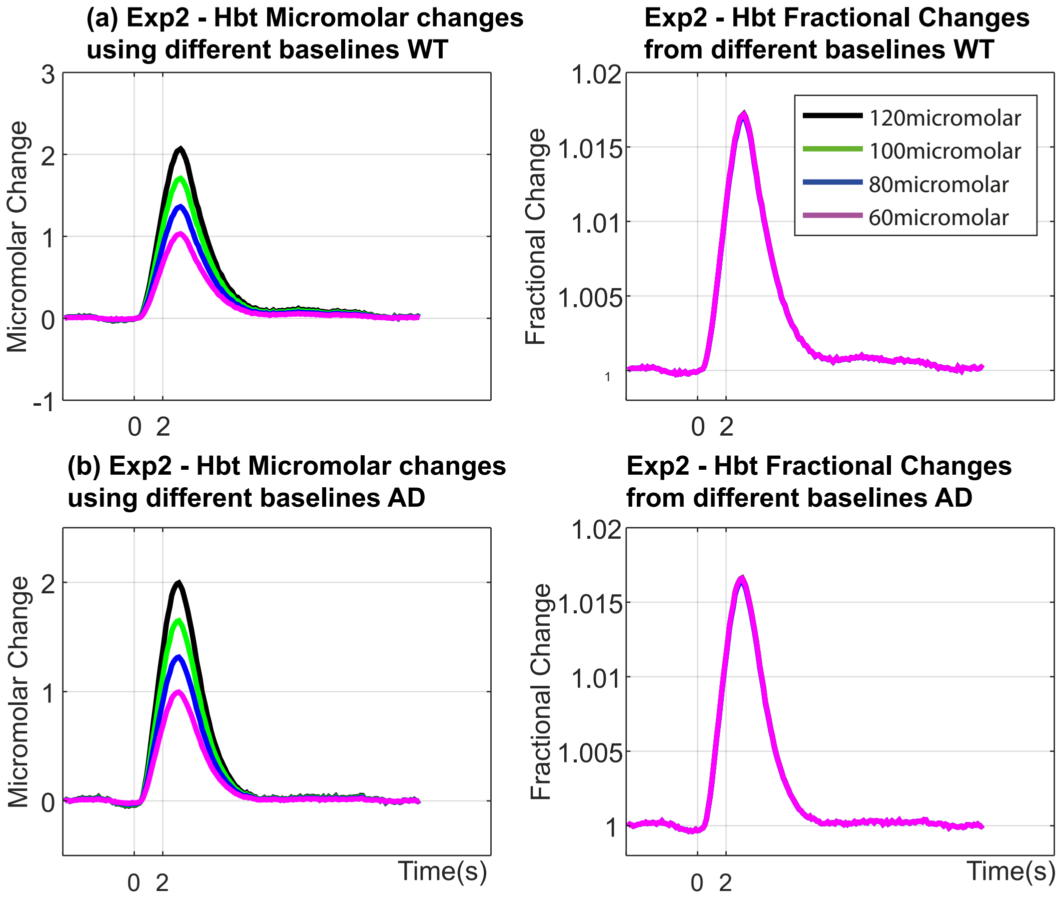

Supplementary Figure 2: Effect on varying baseline blood volume concentration on fractional hemodynamic responses for WT and J20-AD mice. (a) Left hand side micromolar responses using varying baseline WT mice. Right hand side corresponding fractional changes from the varying Hbt baselines. (b) Left hand side micromolar responses using varying baseline J20-AD mice. Right hand side corresponding fractional changes from the varying Hbt baselines.
